# Digital health education needs for women with urinary incontinence in Kigali Rwanda, a phenomenological qualitative study

**DOI:** 10.1371/journal.pgph.0006703

**Published:** 2026-10-08

**Authors:** Alexandre Dukundane, Rachel Murekatete, Jimmy Opiyo, Laura Keyser, Jessica McKinney, Rashidah Nambaziira, Robert Ojiambo

**Affiliations:** 1 University of Global Health Equity, Kigali city, Rwanda; 2 Mama, LLC, Massachusetts, United States of America; University of Washington Seattle Campus: University of Washington, UNITED STATES OF AMERICA

## Abstract

Urinary incontinence (UI), an involuntary loss of urine, is a widespread pelvic floor disorder affecting 20–50% of women worldwide, with a 21% prevalence in Sub-Saharan Africa. Contributing factors include genetics, childbirth, and chronic conditions, leading to significant physical, social, and emotional challenges such as fear of leakage and isolation. Despite its impact, many affected women lack awareness of UI and available treatments, which include medication, surgery, and pelvic floor muscle training (PFMT). Knowledge gaps and misconceptions persist, hindering timely diagnosis and management. In response to these challenges, healthcare systems in Africa are adopting digital technologies (DTs), including WhatsApp and social media, to facilitate patient education and professional collaboration. Studies indicate that digital platforms can support women in managing UI, particularly through PFMT. However, little is known about women’s digital health education needs, barriers, and platform preferences. This study explored these aspects among women with UI in Kigali through a phenomenological qualitative approach across four hospitals: Masaka, Rwanda Military, Kibagabaga, and King Faisal. Using purposive sampling, 20 women aged 20–70 were selected. Data analysis was conducted through thematic analysis using deductive and inductive coding. The findings revealed four key themes. First, there are significant knowledge gaps about UI, necessitating multimodal education to address misinformation and misconceptions. Second, participants regularly use digital devices and the internet, recognizing their benefits and affordability for accessing health information. Third, barriers to digital health education included distrust in the accuracy of online information and financial constraints in maintaining digital devices and internet services. Lastly, participants expressed a strong interest in multimodal digital health education platforms to receive UI-related information. Many women misunderstood UI, often confusing it with aging or other conditions, leading to delayed diagnosis, emotional distress, social withdrawal, financial strain, and reduced quality of life. Although digitally literate, they rarely sought digital health information on UI due to knowledge gaps, financial limitations, and skepticism about online sources. The study underscores the urgent need for accessible and trustworthy digital health education to bridge knowledge gaps, provide support, and enhance UI management among affected women.

## Background

Urinary incontinence (UI) is a common pelvic floor disorder affecting 20–50% of women worldwide [1], with a 21% prevalence in Sub-Saharan Africa [2] and an estimated 42% in Rwanda [3]. UI is categorized into stress UI, urgency UI, and mixed UI, with contributing factors including genetics, age, childbirth, chronic conditions, and lifestyle factors [4]. Women with UI face significant challenges, including fear of leakage, social isolation, and financial burdens associated with management costs [5]. Despite its impact, there is a substantial knowledge gap, with many women unaware of UI, its treatments, and management options [6^].^

Digital health technologies (DTs) present an opportunity to bridge these knowledge gaps, offering education on UI management, including pelvic floor muscle training (PFMT) [7]. While African healthcare systems are increasingly integrating digital solutions such as WhatsApp, blogs, and social media for professional collaboration and patient education, concerns remain regarding the accessibility, scientific validity, and cultural relevance of these platforms [8]. Studies show that digital health solutions have been effective in managing other health conditions in Rwanda, such as diabetes, indicating the potential for UI education and management through digital platforms [9]^.^

This study aims to assess the digital health education needs, facilitators, barriers, and preferred digital health platforms for women with urinary incontinence (UI) in Kigali, Rwanda. Specifically, it seeks to document women’s digital health education needs, identify facilitators and barriers to digital health platform engagement, and determine the most preferred digital platforms for UI education and self-management.

## Methods

### Ethics statement

This research was conducted by respecting ethical standards according to the Declaration of Helsinki (1964). The clearance was granted by the University of Global Health Equity Institutional Review Board [UGHE-IRB/2024/295], King Faisal Hospital Rwanda [KFH/2024/195/IRB], Masaka District Hospital [500/MSK/DH/2024], Kibagabaga Level Two Teaching Hospital [561/HOP.KIBAG/2024], Rwanda Military Hospital [247/RMH/COMDT/2024]. Data collectors out of health facilities of interest were trained on study participants’ rights, confidentiality and ethical norms. Written informed consents were obtained from all study participants before starting interview and data were anonymously collected. The privacy and confidentiality for the study participants were ensured throughout and after the study. No benefits for study participants. Study participants were informed that there was no risk to withdraw from the study.

### Study design and setting

This study employed a qualitative descriptive study design to explore the digital health education needs, facilitators, barriers, and preferred platforms for women with urinary incontinence (UI) in Kigali, Rwanda from 15 June 2024–28 July 2024. The study was conducted across four healthcare facilities: King Faisal Hospital, Rwanda Military Hospital, Masaka Hospital, and Kibagabaga Hospital. These institutions were selected due to their specialized care in gynecology, urology, and women’s health services, ensuring a diverse participant pool and a conducive environment for research.

### Study population and eligibility criteria

The study population comprised women attending outpatient department (OPD) services at the selected hospitals. Inclusion criteria required participants to be between 20 and 70 years old, possess access to personal or shared phones, have basic literacy skills, and report experiencing UTI symptoms in the past three months. Exclusion criteria included women who declined participation, requested monetary compensation, were unable to respond coherently, were bedridden or critically ill, or had UI caused by a fistula.

### Sample size determination

A purposive sampling strategy was employed to select 20 women from the hospitals. The sample size was determined based on the principle of data saturation, where no new themes emerged after a certain number of interviews. This qualitative approach ensured an in-depth exploration of participants’ experiences, needs, and perceptions regarding digital health education for UI management.

### Data collection tools and procedures

A semi-structured interview guide was developed in English and translated into Kinyarwanda. The guide contained open-ended questions on health education needs, digital health facilitators and barriers, and preferred digital platforms. The interview guide was tested by interviewing three women and refined

Four female data collectors proficient in Kinyarwanda and English conducted the interviews to create a comfortable environment for participants. They received a two-day training covering research objectives, interview techniques, and ethical considerations. Participants were recruited from OPD services, and interviews were conducted in private rooms to ensure confidentiality. Informed consent was obtained before data collection, and interviews were audio-recorded for accuracy. The average duration of each interview was 15 minutes. The collected data was transcribed and translated into English for analysis.

### Domains investigated

1. **Health Education Needs:** Participants shared their general access to health information, specific knowledge about pelvic health and UI, and the accessibility of related resources.
2. **Facilitators and Barriers to Digital Health Engagement:** Women’s perceptions of digital platforms such as websites, mobile health (mHealth) applications, and social media were explored.
3. **Desired Digital Health Platforms:** Participants provided insights into preferred features and improvements for digital health solutions in UI management.

### Thematic analysis

Thematic analysis was conducted using both deductive and inductive approaches. Interviews were transcribed verbatim, read repeatedly to build familiarity with the data, and then coded and analyzed manually to identify recurring themes. Meaningful segments of text were labeled with initial codes that captured ideas relevant to the research questions, and these codes were used to develop a codebook that was continuously updated to maintain consistency throughout the process. Themes were derived from clustering the related quotes and each theme was revisited and compared against the full set of transcripts to ensure coherence and clear distinctions between themes. Each theme was then defined and named to reflect its core meaning and relevance to the research questions, and the analytical narrative was constructed by selecting illustrative participant quotes interpreted in light of the research questions and existing literature. Data validation and regular project backups ensured accuracy and reliability.

## Results

### Demographic characteristics of the participants

The Table 1 shows demographic characteristics that were collected from 20 study participants: gravidity (the number of pregnancies experienced), age, parity (the number of children), educational level, and phone ownership. Gravidity from 0 to 10 with an average of 4.35, age ranged from 20 to 70 years, with an average age of 42.60 years, parity also ranged from 0 to 10, with a mean of 3.50. The Table 2. Highlights the phone ownership and the education level. Non-smartphone ownership is at 40%, while smartphone ownership is higher at 60%. The educational levels are primarily low, with 65% having attended primary education, 20% secondary, and 15% tertiary education.

**Table 1:** Demographic characteristics of participants (age, parity, and gravidity).

Variables	Age	Parity	Gravidity
**N**	20	20	20
**Mean**	42.60	3.50	4.35
**Median**	45.00	4.00	4.00
**Std. Deviation**	12.098	2.724	3.083
**Minimum**	20	0	0
**Maximum**	62	9	10

**Table 2:** Smart and non-smart phone ownership.

Variables		Frequency	Percent
**Non-smart phones**	Yes	8	40.0
	No	12	60.0
	**Total**	**20**	**100.0**
**Smartphones**	Yes	12	60.0
	No	8	40.0
	**Total**	**20**	**100.0**
**Educational level**	Primary	13	65.0
	Secondary	4	20.0
	Tertiary	3	15.0
	**Total**	**20**	**100.0**

### Summary of the themes

The study aimed to document digital educational needs, facilitators, barriers, and preferred digital health platforms for women with UI in Kigali. The analysis revealed four key themes: The first theme highlights the critical need for multimodal education to address knowledge gaps as a result of misinformation and myths about UI. The Second theme emphasizes that prior experience with digital technology, understanding its health benefits, and affording necessary devices and internet services facilitate easier access to digital health resources. The third theme identified the barriers women with UI face in accessing digital health education such as distrust of online information and financial constraints related to accessing and maintaining digital devices and internet services. Finally, the fourth theme reveals that women with UI prefer multichannel digital health education and are interested in accessing information both with and without internet connectivity.

#### Theme 1: There are significant knowledge gaps that require multimodal education and community support to address misinformation, misconceptions, and myths about Urinary Incontinence.

The data revealed a significant lack of information regarding UI among participants, who expressed a generally low level of understanding of UI, causes, symptoms, and available treatment options. Some participants mistakenly linked to Fistula. When interviewing participants about whether they have a UI condition or not, they could first deny but after a proper explanation, they would agree that they have the condition but lacked a proper understanding about it. Initially, women were unaware that their condition could be treated in various ways. They believed they could manage it on their own without needing medical treatment and thought their ways of managing the condition were sufficient. They felt they were managing the condition well and didn’t seek information either digitally, or from the health care workers regarding available treatments. Some women believed that leaking urine was linked to other conditions and diseases, such as diabetes or unsuccessful gynecological surgery. Others were unaware that experiencing urine leakage during activities like coughing, sneezing, or laughing was UI. This lack of knowledge meant they often overlooked or misunderstood these symptoms, not realizing they were signs of a treatable medical condition.

*“I don’t know, I think in real life I don’t have any specific information on the condition.” -* ***43 years old from Rwanda Military Hospital, KA02.***

*“Urinary incontinence? I have some information about it, but I’m not sure about its causes. I understand the condition, but I’m not aware of its specific causes”.* ***-26 years old from Rwanda Military Hospital, KA02.***

*“There are no treatments available”. -* ***51 years old from Masaka Hospital, MA08.***

*“I went to get tested to see if it was diabetes, but they didn’t find it. So, I thought maybe it was because I am getting older like other older people, but it continued****….*** *and I usually thought it happens to someone who had surgery that went wrong, and then the urine starts leaking****” -38 Years Old from Masaka Hospital, MA03.***

***“****Another benefit is that I didn’t realize coughing and experiencing leakage were a problem. Now that I know, I can encourage my friends and family who might have this issue to start addressing it immediately”.* ***-39 years from Rwanda Military Hospital, KA01.***

The study participants also revealed deep-seated misconceptions and myths surrounding UI as many women viewed UI as an inevitable and normal consequence of aging or childbirth leading them to perceive it as an irreversible condition to accept rather than actively seek ways to alleviate its impact on daily life. This belief, coupled with the stigma associated with UI, often prevents women from seeking help or discussing their symptoms openly. This wrong interpretation of the condition results in inappropriate approaches to addressing the condition and seeking proper treatment.

“*I didn’t think of getting treatment because I thought UI was something normal and could happen to everyone.****”-24 years from Masaka Hospital, MA05.***

*“I never went to the hospital because I felt like everyone had that disease, and I thought it was something everyone suffered from.* ***-24 years from Masaka Hospital, MA05.***

*“Many of my friends’ moms are like that; they can’t control their urine. We all just learn to live with it. There is nothing we can do”* 42-year-old ***woman from Kibagabaga Hospital, KI01***.

*“In Rwandan culture, women often feel ashamed of their health issues and are afraid to ask the doctor for help” -* ***57 years old from King Faisal Hospital, KF03.***

Participants often felt lost and unsure about where to seek help for UI due to limited access to reliable information. While some received information on fistula treatment, awareness about managing and treating UI was lacking. They believed that community awareness campaigns would be crucial for spreading accurate information and encouraging women to seek treatment. Effective communication through community health workers was highlighted as essential for providing timely, clear, and accessible information to help individuals manage UI and take appropriate action.

*“I wonder if I should consult a doctor or a specialist to find out more” -* ***42-year-old from Rwanda Military Hospital, KA003.***

*“It just happens; I haven’t heard where it’s treated. I hear on the radio about those who had this problem from surgery gone wrong* ***” -38-year-old Masaka Hospital, MA03.***

*“Only if health workers visit them in the community. Many people in rural areas are unaware of these things (UI).” -* ***70 years old from Masaka Hospital, MA06.***

*“Yeah, if I get an opportunity to get information on the treatment, I will use the pieces of advice given so that I get better”. -* ***24 years old from Masaka Hospital, MA05.***

“*Communication should be the top priority. When there is effective communication, people receive enough information and get help for any problem promptly including UI”****-39 years old from Rwanda Military Hospital, KA01.***

Women suffering from UI have minimal interaction with each other, which significantly limits the exchange of valuable knowledge and support, increasing the sense of isolation and helplessness that many of these women feel. The need for communal support came out as an important strategy to get information concerning health-related issues, saying that such platforms provide a supportive environment, allowing women to share experiences and coping strategies. For example, a participant mentioned that she felt relieved and comforted to know that many other women are also open to talking about the condition, giving her the confidence to openly discuss her experiences and seek more information and support.

*“We should establish clubs where everyone can get to know each other and share about our condition* ***”- said a 45-year-old woman from Masaka Hospital. MA06.***

*“People in the concerned age group must receive advice that urinary incontinence is not a normal part of life but a treatable condition. This advice should be given generally, as many people experience it but may not talk about it.”*-**52-year-old patient from Rwanda Military Hospital**

*“Now, knowing that many people deal with it encourages me to see a doctor and feel comfortable speaking out about my condition”.* ***-24 years from Masaka Hospital, MA05***

They mentioned coping mechanisms such as limiting their water intake, carrying spare clothing for accidents, and avoiding social activities to prevent embarrassment. Participants consistently described how UI disrupts their daily lives. The fear of leakage and the need for frequent bathroom visits cause many women to withdraw from social events and limit their physical activities. This avoidance not only impacts their physical health but also leads to feelings of isolation and emotional distress.

*“Urinary incontinence has really turned my life upside down, leaving me struggling financially and emotionally. Dealing with this condition has been incredibly tough. Imagine having to change four pads in just 20 minutes—it’s not just inconvenient, but also expensive. It’s made me feel like I can’t fully participate in social activities because I’m always worried about accidents. This constant fear of leaks has forced me to constantly carry extra clothes and underwear, which has been both embarrassing and exhausting. Coping with this for six months has been overwhelming, and finding a way to manage it has been a real challenge for me”* ***-46-year-old Rwanda Military Hospital KA 2314.***

*“It’s concerning because I worry about what will happen if I’m with people and can’t find a toilet. Although I’m advised to drink a lot of water due to my health condition, I avoid doing so when traveling, such as going from Kirehe to Kigali, because it’s difficult to find a toilet along the way..”* ***King Faisal Hospital, KF01***

*“It has changed. If it happens when you are far from home without spare clothes or a place to wash, it can be very uncomfortable and embarrassing. " -* ***48 years old Rwanda Military Hospital 0018.***

*When I go out, I avoid drinking water. If I must go to morning prayers, I can’t drink much beforehand or on the way. You know your own body if you have a condition. During festivals, I also drink very little.”* ***-38 years old Rwanda Military Hospital KA0100.***

#### Theme 2: Participants regularly use digital devices and the internet, acknowledging its value, benefits, and affordability as a facilitator to access digital health information.

Most participants demonstrated a reasonable level of digital literacy, regularly using these tools to research various health issues. They showed confidence in seeking information about common health concerns such as headaches or COVID-19 prevention, indicating a proactive approach to health management through digital health information.

*“Well, one can use digital platforms for health information. For instance, if you have a headache, you might wonder what the cause is. Or if you have a stomach problem, you might try to find out exactly what’s causing it.” -* ***26 years old from Rwanda Military Hospital KA004.***

*“During Covid, I looked for ways to protect myself and found information on how to prevent Covid.”-* ***26 years old from Rwanda Military Hospital, KA004.***

Some participants found it beneficial to educate themselves about their conditions using digital health information sources before consulting healthcare professionals. Having prior knowledge from these resources enabled them to understand their condition better, engage effectively with doctor’s advice, and make informed decisions as they mentioned various previous platforms they used to educate themselves about different health conditions, including online magazines, and social media groups, where they find information.

*" I’ve done a lot of research on health issues, especially on the internet, before deciding to see a doctor. I made sure to educate myself about the disease first.” -* ***57 years old from King Faisal Hospital, KF03.***

*" For example, if you have a headache, you might wonder about the cause. Or if you have a stomach problem, you try to find out exactly what is causing it.* ***”-26 years old Rwanda Military Hospital, KA004.***

*“Yes, I use technology to get information on different health conditions like WhatsApp, I also read magazines on the internet.* ***”-42 Years Old Rwanda Military Hospital, KA003.***

Participants acknowledged the benefits and importance of using digital and internet technology to access health information. They confirmed that digital health information is valuable because it can be accessed from anywhere and anytime, making it useful no matter the location. Some participants mentioned that it’s not expensive, highlighting how convenient and effective digital platforms are in managing health, and noting how they have made it easier for her to stay informed.

*“The benefit is that technology can reach different people because many are unaware of the UI condition,”* ***said a 45-year-old patient at Masaka Hospital, MA06.***

*“Yes, there are some strategies that have helped me. For example, I read an article about ways to stay awake at work, which can be useful for people who tend to fall asleep while sitting down. The first benefit is that I don’t waste time. I don’t spend much time waiting at the doctor’s office. Additionally, I gain more knowledge, which I can share with my colleagues* ***.”-48 years old from Rwanda Military Hospital, KA0018.***

*It’s just to give you that information. Providing you with the necessary information, yeah is necessary and inexpensive, and does not take up your time, it is something that you can look at while sitting somewhere on the bus and monitor the information and help yourself* ***" -26 years old from Rwanda Military Hospital, KA004.***

Participants reported having access to digital health information through various means, including SMS, phone calls, and internet access. Many use data bundles and WiFi, mentioning they can afford to purchase MBs and use them when not working while relying on WiFi at work. Their previous experience with the internet and consistent WiFi access enables them to use online resources daily, making digital platforms an integral part of the way of receiving health information.

*“The way I can receive information is the way I receive messages now. For now, they used to send messages, when you get tested for diabetes, you know, or cervical cancer. They take your phone number and send you a message about that disease, they send you a message on how you can prevent it, and how to treat it, and you immediately see it. “Yes, that’s what I think would be easier for me, “* ***said a 45-year-old patient at Masaka Hospital, MA06.***

*“I use MBS at home, and I use Wi-Fi at work.”* ***42 years old, Kibagabaga Hospital KA01.***

*“Eh, I use it all. At home, I use WiFi, and at work, I use mobile data (MBs).” -* ***42 years old, Rwanda Military Hospital, KA003.***

#### Theme 3: Women with UI face barriers in accessing digital health education due to distrust of online information’s accuracy and financial constraints to maintain digital devices and internet services.

Doubts about online health information, particularly on social media came out as a barrier to using digital information as participants cited concerns about encountering false or misleading advice leading to disengagement with digital health resources. Some women mentioned that the limited availability of specific health information online leads them to prefer consulting with healthcare professionals directly.

*“Sometimes people post false information on social media. When I realize it’s a lie, it makes me unhappy. I don’t like the fake things people share online.”* ***-48 years old from Rwanda Military Hospital, KA0018.***

*When it’s necessary, I feel it’s much better than relying on online platforms. On social media, the information might be intended to help, but I might encounter an urgent health issue and the information might not be available yet. In such cases, seeing a doctor directly is more reliable.”-* ***39-year-old Rwanda Military Hospital, KA01.***

While all the women who participated in this study had phone access, not all of them owned smartphones. This means that some women don’t have access to digital information that requires internet connectivity. For those facing financial constraints, buying a more advanced phone, or repairing damaged devices may not be feasible, which limits their ability to access a wide range of digital health information online. The participants also reported the difficulty in affording mobile data (megabytes) needed to access online platforms. They expressed a belief that free access to these resources would encourage wider usage among individuals who currently face financial challenges.

“*It’s that smartphones are expensive, and all people can’t afford to buy them unless they are affordable”.-* ***45 years old from Masaka Hospital, MA04.***

*“Yes, when my phone fell into the water, I took it to a technician and was able to reuse it. However, once when I was in the toilet, my phone dropped out of my pocket into a 15-meter-deep toilet, and I had to leave it there.”-* ***50 years old from Rwanda Military Hospital, KA20B.***

*“One major challenge is that a person has to spend money to access it. Finding money to buy those megabytes can be difficult. If it were free, everyone would be encouraged to use it.* ***’38 years old from Masaka Hospital, MA03***

#### Theme 4: Women experiencing Urinary Incontinence (UI) showed interest in accessing information from multimodal digital health education platforms.

During our study, we found that women prefer to access information about UI in two main ways. Some favor non-internet methods like SMS, calls, radio, and TV. Radio broadcasts were particularly popular as an effective way to reach a broad audience who are not online, ensuring wider access to important health information.” Text messages were also favored for their convenience in providing timely updates on prevention and treatment. Other women liked receiving information through social media platforms that use the internet like WhatsApp, websites, and apps.

*“Campaigning through various channels can be very effective. Announcements on radio, television, and newspapers can raise awareness… hospitals, especially those that follow up on maternal health, can provide information and counseling about bladder leakage. Often, because the condition doesn’t cause pain, people don’t take it seriously, so these efforts can help highlight it.* ***said a 45-year-old patient at Masaka Hospital, MA06.***

*"I think the easiest way is to broadcast it on the radio during certain hours because many people enjoy listening to the radio.” -* ***52 years old from Rwanda Military Hospital, KA21.***

She added, *“Text messages are the best because they allow me to get information without spending a lot of time going to the hospital.”* ***-52 years old from Rwanda Military Hospital KA21*.**

*“The best way for me to receive information is through text messages, just like I do now. For example, when you get tested for diabetes or cervical cancer, they take your phone number and send you messages about the disease. These messages include information on prevention and treatment, and I can see them immediately. Yes, that’s what I think would be easiest for me”.* ***46 years old from Masaka Hospital, MA06.***

*“Perhaps broadcasting it through radio or television would be better.”-* ***57 years old from King Faisal Hospital, KF02.***

*When discussing the internet, one might argue that the ease of accessing information depends on the platform—whether it’s WhatsApp, Twitter, or others.* ***42 years old, Kibagabaga Hospital, KI22.***

*“Using the telephone and the internet is easier than spending time going to the doctor or healthcare workers. For me, the internet is much more convenient than any other option.”3* ***9 years old from Military Hospital, KA0***

## Discussion

The study aimed to explore digital educational needs, barriers, and preferences among women with UI in Kigali. The findings highlight significant knowledge gaps, wide digital access, and usage for general health issues, barriers such as distrust of online information and financial constraints, and a strong preference for multimodal digital health education.

### The Necessity of Multimodal Digital Health Education to Dispel Misinformation and Myths About UI

Our study highlights substantial gaps in participants’ knowledge about UI. Many women did not understand UI, its types, symptoms, and treatment options. Similarly, a study in Brazil found that 46.3% of women had never heard of UI, and 66.1% did not know about medical treatments [10].This knowledge gap can lead to delayed diagnosis and treatment [11].

The study revealed widespread misconceptions and myths about UI, with many participants believing it was a normal part of aging, a finding consistent with similar beliefs documented in a U.S. nursing home study [12].

Shame and embarrassment emerged as common barriers, as cultural and social norms discouraged open discussions about reproductive health, mirroring findings from Spain where stress and shame were further compounded by misinformation and myths surrounding UI [13]. Creating safe environments for discussion and using digital platforms to break stigma could help. Studies suggest that digital health education can improve understanding and encourage open conversations [14].

Our study found that UI significantly impacts quality of life, leading to social withdrawal, limited physical activities, financial strain, and emotional distress. The constant fear of leakage and frequent bathroom visits resulted in isolation and loneliness. Financially, UI management, including the cost of incontinence products, was burdensome. Research has shown that the high cost of incontinence products makes them unaffordable for many [15]. These economic and social burdens contribute to poor mental health outcomes, similar to findings in previous studies [16]. Unique cultural challenges in Rwanda, such as reluctance to discuss reproductive health publicly, further hinder efforts to address UI. Culturally sensitive educational approaches, such as digital health interventions, may provide a solution.

### The role of digital devices and the internet in facilitating access to health information

Digital health literacy is crucial for effectively seeking, understanding, and using online health information. It involves technical skills, information literacy, and health literacy [17]. Our study showed that participants were familiar with using digital devices and the internet to research general health issues but rarely searched for information about UI. This highlights the need for targeted digital education to encourage UI-related information-seeking.

Participants trusted digital platforms for common health concerns, suggesting they would be open to using them for UI education if relevant content were available. A study in Uganda showed that mobile applications enhanced sexual and reproductive health knowledge and service utilization [18]. Digital health interventions should consider factors that facilitate public engagement, such as motivation, platform quality, and relevance [19]

Women in our study valued digital platforms for quick access to health information. A study in Nigeria found that widespread access to information and communication technologies (ICT) could improve healthcare outcomes [20]. Similarly, research indicates that women find online health information valuable and trustworthy [21]. Digital platforms enable rapid information-sharing, empowering communities with knowledge. Previous studies suggest that digital health technology can boost confidence in managing health and aid preventive care [22].

Midwives and women in the Middle East, including Syrian refugees, viewed digital technology as cost-effective and beneficial for enhancing knowledge about family planning [23]. These findings suggest that integrating UI education into existing digital health initiatives could be effective.

Our study found that participants had daily internet access through mobile phones, using WiFi at work and mobile data elsewhere. Affordable mobile data facilitated regular internet use, ensuring constant connectivity. Previous research indicates that digital interventions for UI are affordable and can improve treatment outcomes [24].

### Barriers to digital health education: Distrust and financial constraints

A key barrier identified was distrust of online health information. Participants reported encountering misleading or conflicting advice, making them hesitant to rely on digital sources. This aligns with studies showing that misinformation can hinder engagement with digital health education [25]. Addressing these concerns requires credible sources and expert-reviewed content.

Financial constraints also posed a significant challenge. Some participants struggled with the cost of internet access and mobile data, limiting their ability to access digital health resources. Research has shown that financial barriers significantly impact health information access [26]. Providing subsidized or free access to UI education platforms could help overcome this barrier.

## Conclusion

This study excluded illiterate individuals, younger women under 20, and older women over 70, limiting insights into their unique experiences with UI. Conducting the study solely in urban Kigali restricted findings to an urban setting, excluding rural perspectives on healthcare access and digital resources. Future research should include diverse age groups, literacy levels, and geographic settings for a more comprehensive understanding of UI. Healthcare authorities should launch community-based educational programs through TV, radio, social media, and outreach to dispel UI myths and provide accurate information. Hospitals and community centers should distribute easy-to-understand materials and establish support groups. Training healthcare providers to discuss UI during routine visits will improve awareness and treatment access.

Digital health platforms should offer trustworthy, accessible UI resources. Software Developers, particularly, should create verified online tools. Educational campaigns must enhance digital literacy, helping women find credible health information. For those without internet access, SMS and phone-based updates can provide essential UI-related support. Online forums and support groups should be promoted to foster a community for women managing UI. Future research should include diverse age groups, literacy levels, and geographic settings for a more comprehensive understanding of UI.

## Abbreviations

UGHE: University of Global Health Equity
IRB: Institutional Review Board
UI: Urinary Incontinence
FI: Fecal Incontinence
PFD: Pelvic Floor Disorders
PFMT: Pelvic Floor Muscle Training
WHO: World Health Organization
DTs: Digital Therapeutics
HCD: Human-Centered Design
POP: Pelvic Organ Prolapse
SSA: Sub-Saharan Africa
UUI: Urgency Urinary Incontinence
SUI: Stress Urinary Incontinence
OAB: Overactive Bladder
BMI: Body Mass Index
QoL: Quality of Life
ICU: Intensive Care Unit
3IQ: Three Intelligent Questions
OPD: Outpatient Department
PIs: Principal Investigators
PFME: Pelvic Floor Muscle Exercises
