# Supplementary material for: Digital health education needs for women with urinary incontinence in Kigali Rwanda, a phenomenological qualitative study
Source: PLOS Glob Public Health. 2026 Oct 8;6(10):e0006703. doi: 10.1371/journal.pgph.0006703 (PMC13648990; doi:10.1371/journal.pgph.0006703)
Supplement: Supplementary file 1 [file pgph.0006703.s002.docx]

June 04, 2026

Manuscript ID: PGPH-D-26-00970

Dear Editors,

It is our pleasure to submit to you the revised article titled **Digital Health Education Needs for Women with Urinary Incontinence in Kigali Rwanda, a phenomenological qualitative study***.* We have carefully reviewed the thoughtful reviewer comments and have revised accordingly.

Below you will find the point-by-point response to reviewers’ comments. We also submit a clean copy of the Main Document and the highlighted copy of the Main Document of the revised article.

We thank you very much for your time and for your consideration of this research for publication in PLOS Global Public Health.

Sincerely,

Alexandre Dukundane, MD, MSc, University of Global Health Equity, Rwanda

Response to editors for the article, “**Digital Health Education Needs for Women with Urinary Incontinence in Kigali Rwanda, a phenomenological qualitative study**”

| **#** | | **Editor comments** | **Response** |
| --- | --- | --- | --- |
| **Editor(s)' Comments to Author** | | | |
| 1 | Please provide additional details regarding participant consent. In the ethics statement in the Methods and online submission information, please ensure that you have specified (1) whether consent was informed and (2) what type you obtained (for instance, written or verbal, and if verbal, how it was documented and witnessed). If your study included minors, state whether you obtained consent from parents or guardians. If the need for consent was waived by the ethics committee, please include this information.  If you are reporting a retrospective study of medical records or archived samples, please ensure that you have discussed whether all data were fully anonymized before you accessed them and/or whether the IRB or ethics committee waived the requirement for informed consent. If patients provided informed written consent to have data from their medical records used in research, please include this information. | | Thank you for your comment. We have clarified in the manuscript that written informed consent was obtained from all participants prior to their involvement in the study. Please refer to line 91 of the revised manuscript. |
| 2 | Please amend your detailed Financial Disclosure statement. This is published with the article. It must therefore be completed in full sentences and contain the exact wording you wish to be published. | | Thank you for your comment. The financial disclosure statement has been included in the manuscript. Please refer to lines 533–534 of the revised manuscript. |
| 3 | Please ensure that your Ethics Statement is available in its entirety at the beginning of your Methods section, under a subheading 'Ethics Statement'. | | Thank you for your comment. The ethics statement has been moved to the beginning of the Methods section. Please refer to the revised manuscript. |
| **Additional Editor Comments** | | | |
| 1 | The study design: The authors indicated that they adopted a phenomenological approach to the study. After a thorough read of the manuscript, I would disagree that what the authors have described qualifies as a phenomenological study. In phenomenology, the participant selected must have experienced a particular phenomenon such as near death, cancer remission, losing a partner or a child. Notice that these phenomena carry deep emotional and psychological emersion on the part of the respondent. I would argue, therefore, that doing a Qualitative descriptive study would be the most appropriate research design for this study. See the two resources below:  Colorafi KJ, Evans B. Qualitative Descriptive Methods in Health Science Research. HERD. 2016 Jul;9(4):16-25. doi: 10.1177/1937586715614171. Epub 2016 Jan 19. PMID: 26791375; PMCID: PMC7586301.  https://oercollective.caul.edu.au/qualitative-research/chapter/5/ | | Thank you for your valuable comment. After reviewing the resources and references you provided, we updated the study design to better align with the recommended approach. Please see line 96 of the revised manuscript. |
| 2 | Data analysis approach: My point one leads to point two. Usually when a researcher adopts a phenomenological study design, for epistemological consistency, interpretative phenomenological analysis (IPA) is also adopted. This is not the case with this paper and I wonder why. IPA, rooted in phenomenology and hermeneutics, focuses on deeply exploring personal lived experiences within small, homogeneous samples. The authors should consider providing a rationale for not adopting the interpretive phenomenological analysis approach or should as I proposed above, change this design to a qualitative descriptive study design which aligns with the data analysis approach that they described. This point then leads to my third concern. | | Thank you for your valuable comment. After reviewing the resources and references you provided, we updated the study design to better align with the recommended approach. Please see line 96 of the revised manuscript. |
| 3 | Whitewash description of data analysis: I would have loved to see a more detailed description of the data analysis approach. Simply stating that one did a thematic qualitative approach to analysis is not good enough. I will encourage the authors to describe in detail or step by step how they did this thematic analysis. My steps have been proposed in the literature and it will be great to see that one of these steps are taken narrations of how they were clearly applied in this study. | | Thank you for your comment. To enhance clarity and transparency, we have expanded the description of the thematic analysis methodology. Details can be found in lines 137–147 of the revised manuscript. |
| **Reviewer 1 comments** | | | |
| 1 | | The objective of the study is too broad and needs to be divided so as to align with the specific themes used in the discussion of the findings. Suggestion introduces the specific sub objectives that align with the specific thematic areas in the discussion. | Thank you for your comment. We have summarized and reorganized the study objectives into a general objective and specific objectives. Please refer to the revised manuscript for the updated section. |
| 2 | | In line 434 there is the assertion that the study found that UI significantly impacts quality of life, this assertion of significance is harder to justify for a qualitative study as there are no specific tests of significance that can be carried out. This needs to be reviewed. | Thank you for your comment. We reviewed the line and rephrased the entire paragraph accordingly. Please refer to the revised manuscript. |
| **Reviewer 2 comments** | | | |
| 1 | | Overall, the paper is well written. However, there is a concern regarding the wide age variation among participants. The likelihood and underlying causes of urinary incontinence in a 20-year-old woman may differ substantially from those in a 62-year-old woman. Including such a broad age range may affect the comparability of findings. A narrower age range, would likely improve the validity and interpretability of the results. | Thank you for this thoughtful observation. We acknowledge the reviewer's concern regarding the wide age range of participants (20–62 years) and recognize that the etiology, prevalence, and lived experience of urinary incontinence (UI) do indeed vary across different life stages.  However, it is important to note that this study employed a qualitative descriptive design, which prioritizes depth of understanding over statistical comparability. The aim was not to compare UI experiences across age groups but rather to explore the digital health education needs, facilitators, and barriers as experienced by women with UI in Kigali, Rwanda, regardless of age.  Furthermore, the inclusion of women across a wide age range strengthens the transferability of the findings, as it reflects the real-world diversity of women affected by UI in the Rwandan context. |
