# Supplementary material for: Digital health education needs for women with urinary incontinence in Kigali Rwanda, a phenomenological qualitative study
Source: PLOS Glob Public Health. 2026 Oct 8;6(10):e0006703. doi: 10.1371/journal.pgph.0006703 (PMC13648990; doi:10.1371/journal.pgph.0006703)
Supplement: Peer review history

# Peer review: Decision Letter 0

**DOI:** 10.1371/journal.pgph.0006703.r001

**Authors:** Ferdinand Mukumbang

10 May 2026

PGPH-D-26-00970

Digital Health Education Needs for Women with Urinary Incontinence in Kigali Rwanda, a phenomenological qualitative study

PLOS Global Public Health

Dear Dr. Dukundane,

Thank you for submitting your manuscript to PLOS Global Public Health. After careful consideration, we feel that it has merit but does not fully meet PLOS Global Public Health’s publication criteria as it currently stands. Therefore, we invite you to submit a revised version of the manuscript that addresses the points raised during the review process.

I invite you to address the comments that both reviewers provided including the one that I provided on the study design and methodology.

Please submit your revised manuscript by June 30, 2026. If you will need more time than this to complete your revisions, please reply to this message or contact the journal office at globalpubhealth@plos.org. When you’re ready to submit your revision, log on to https://www.editorialmanager.com/pgph/ and select the 'Submissions Needing Revision' folder to locate your manuscript file.

Please include the following items when submitting your revised manuscript:

- A letter that responds to each point raised by the editor and reviewer(s). You should upload this letter as a separate file labeled 'Response to Reviewers'.
- A marked-up copy of your manuscript that highlights changes made to the original version. You should upload this as a separate file labeled 'Revised Manuscript with Track Changes'.
- An unmarked version of your revised paper without tracked changes. You should upload this as a separate file labeled 'Manuscript'.

Guidelines for resubmitting your figure files are available below the reviewer comments at the end of this letter.

As the corresponding author, your ORCID iD is verified in the submission system and will appear in the published article. PLOS supports the use of ORCID, and we encourage all coauthors to register for an ORCID iD and use it as well. Please encourage your coauthors to verify their ORCID iD within the submission system before final acceptance, as unverified ORCID iDs will not appear in the published article. *Only*  the individual author can complete the verification step; PLOS staff *cannot*  verify ORCID iDs on behalf of authors.

We look forward to receiving your revised manuscript.

Kind regards,

Ferdinand C Mukumbang, PhD

Academic Editor

PLOS Global Public Health

**Journal Requirements:**

1. Please provide additional details regarding participant consent. In the ethics statement in the Methods and online submission information, please ensure that you have specified (1) whether consent was informed and (2) what type you obtained (for instance, written or verbal, and if verbal, how it was documented and witnessed). If your study included minors, state whether you obtained consent from parents or guardians. If the need for consent was waived by the ethics committee, please include this information.

If you are reporting a retrospective study of medical records or archived samples, please ensure that you have discussed whether all data were fully anonymized before you accessed them and/or whether the IRB or ethics committee waived the requirement for informed consent. If patients provided informed written consent to have data from their medical records used in research, please include this information.

2. Please amend your detailed Financial Disclosure statement. This is published with the article. It must therefore be completed in full sentences and contain the exact wording you wish to be published.

i. Please clarify all sources of financial support for your study. List the grants, grant numbers, and organizations that funded your study, including funding received from your institution. Please note that suppliers of material support, including research materials, should be recognized in the Acknowledgements section rather than in the Financial Disclosure.

ii. State the initials, alongside each funding source, of each author to receive each grant. For example: "This work was supported by the National Institutes of Health (####### to AM; ###### to CJ) and the National Science Foundation (###### to AM)."

iii. State what role the funders took in the study. If the funders had no role in your study, please state: “The funders had no role in study design, data collection and analysis, decision to publish, or preparation of the manuscript.”

iv. If any authors received a salary from any of your funders, please state which authors and which funders.

3. Please ensure that your Ethics Statement is available in its entirety at the beginning of your Methods section, under a subheading 'Ethics Statement'.

If the reviewer comments include a recommendation to cite specific previously published works, please review and evaluate these publications to determine whether they are relevant and should be cited. There is no requirement to cite these works unless the editor has indicated otherwise.

**Additional Editor Comments (if provided):**

I applaud the authors for such a well-written paper, which has allowed the reviewers to read and provide their comments. In addition to the comments that the two reviewers have provided, here are some additional considerations:

(1) The study design: The authors indicated that they adopted a phenomenological approach to the study. After a thorough read of the manuscript, I would disagree that what the authors have described qualifies as a phenomenological study. In phenomenology, the participant selected must have experienced a particular phenomenon such as near death, cancer remission, losing a partner or a child. Notice that these phenomena carry deep emotional and psychological emersion on the part of the respondent. I would argue, therefore, that doing a Qualitative descriptive study would be the most appropriate research design for this study. See the two resources below:

Colorafi KJ, Evans B. Qualitative Descriptive Methods in Health Science Research. HERD. 2016 Jul;9(4):16-25. doi: 10.1177/1937586715614171. Epub 2016 Jan 19. PMID: 26791375; PMCID: PMC7586301.

https://oercollective.caul.edu.au/qualitative-research/chapter/5/

(2) Data analysis approach: My point one leads to point two. Usually when a researcher adopts a phenomenological study design, for epistemological consistency, interpretative phenomenological analysis (IPA) is also adopted. This is not the case with this paper and I wonder why. IPA, rooted in phenomenology and hermeneutics, focuses on deeply exploring personal lived experiences within small, homogeneous samples. The authors should consider providing a rationale for not adopting the interpretive phenomenological analysis approach or should as I proposed above, change this design to a qualitative descriptive study design which aligns with the data analysis approach that they described. This point then leads to my third concern.

(3) Whitewash description of data analysis: I would have loved to see a more detailed description of the data analysis approach. Simply stating that one did a thematic qualitative approach to analysis is not good enough. I will encourage the authors to describe in detail or step by step how they did this thematic analysis. My steps have been proposed in the literature and it will be great to see that one of these steps are taken narrations of how they were clearly applied in this study.

[Note: HTML markup is below. Please do not edit.]

Reviewers' comments:

Reviewer’s Responses to Questions

**Comments to the Author**

1. Does this manuscript meet PLOS Global Public Health’s publication criteria?

Reviewer #1: Yes

Reviewer #2: Yes

**********

2. Has the statistical analysis been performed appropriately and rigorously?

Reviewer #1: N/A

Reviewer #2: Yes

**********

3. Have the authors made all data underlying the findings in their manuscript fully available (please refer to the Data Availability Statement at the start of the manuscript PDF file)?

The PLOS Data policy

Reviewer #1: No

Reviewer #2: Yes

**********

4. Is the manuscript presented in an intelligible fashion and written in standard English?

Reviewer #1: Yes

Reviewer #2: Yes

**********

**Reviewer #1:**  The paper is interesting to read. However, there are a few thigs that need to be adjusted.

1. The objective of the study is too broad and needs to be divided so as to align with the specific themes used in the discussion of the findings. Suggestion introduce the specific sub objectives that align with the specific thematic areas in the discussion.

2. In line 434 there is the assertion that the study found that UI significantly impacts quality of life, this assertion of significance is harder to justify for a qualitative study as there are no specific tests of significance that can be carried out. This needs to be reviewed.

**Reviewer #2:**  Overall, the paper is well written. However, there is a concern regarding the wide age variation among participants. The likelihood and underlying causes of urinary incontinence in a 20-year-old woman may differ substantially from those in a 62-year-old woman. Including such a broad age range may affect the comparability of findings. A narrower age range, would likely improve the validity and interpretability of the results.

**********

what does this mean?). If published, this will include your full peer review and any attached files.

**Do you want your identity to be public for this peer review?**  If you choose “no”, your identity will remain anonymous but your review may still be made public.

For information about this choice, including consent withdrawal, please see our Privacy Policy

Reviewer #1: No

Reviewer #2: No

**********

[NOTE: If reviewer comments were submitted as an attachment file, they will be attached to this email and accessible via the submission site. Please log into your account, locate the manuscript record, and check for the action link "View Attachments". If this link does not appear, there are no attachment files.]

**Figure Resubmissions:**

# Author comment: Author response to Decision Letter 1

**DOI:** 10.1371/journal.pgph.0006703.r002

4 Jun 2026

Attachment Submitted filename: Response to reviewers.docx

# Peer review: Decision Letter 1

**DOI:** 10.1371/journal.pgph.0006703.r003

**Authors:** Ferdinand Mukumbang

23 Sep 2026

Digital Health Education Needs for Women with Urinary Incontinence in Kigali Rwanda, a phenomenological qualitative study

PGPH-D-26-00970R1

Dear Alexandre Dukundane,

We are pleased to inform you that your manuscript 'Digital Health Education Needs for Women with Urinary Incontinence in Kigali Rwanda, a phenomenological qualitative study' has been provisionally accepted for publication in PLOS Global Public Health.

Before your manuscript can be formally accepted you will need to complete some formatting changes, which you will receive in a follow up email. A member of our team will be in touch with a set of requests.

Please note that your manuscript will not be scheduled for publication until you have made the required changes, so a swift response is appreciated.

IMPORTANT: The editorial review process is now complete. PLOS will only permit corrections to spelling, formatting or significant scientific errors from this point onwards. Requests for major changes, or any which affect the scientific understanding of your work, will cause delays to the publication date of your manuscript.

If your institution or institutions have a press office, please notify them about your upcoming paper to help maximize its impact. If they'll be preparing press materials, please inform our press team as soon as possible -- no later than 48 hours after receiving the formal acceptance. Your manuscript will remain under strict press embargo until 2 pm Eastern Time on the date of publication. For more information, please contact globalpubhealth@plos.org.

Thank you again for supporting Open Access publishing; we are looking forward to publishing your work in PLOS Global Public Health.

Best regards,

Ferdinand C Mukumbang

Academic Editor

PLOS Global Public Health

***********************************************************

Reviewer Comments (if any, and for reference):

Reviewer’s Responses to Questions

**Comments to the Author**

Reviewer #1: All comments have been addressed

**********

publication criteria?

Reviewer #1: Yes

**********

3. Has the statistical analysis been performed appropriately and rigorously?

Reviewer #1: Yes

**********

4. Have the authors made all data underlying the findings in their manuscript fully available (please refer to the Data Availability Statement at the start of the manuscript PDF file)?

The PLOS Data policy

Reviewer #1: No

**********

5. Is the manuscript presented in an intelligible fashion and written in standard English?

Reviewer #1: Yes

**********

Reviewer #1: I have no other issues. All issues addressed.

**********

what does this mean?). If published, this will include your full peer review and any attached files.

**Do you want your identity to be public for this peer review?**  If you choose “no”, your identity will remain anonymous but your review may still be made public.

For information about this choice, including consent withdrawal, please see our Privacy Policy

Reviewer #1: No

**********
